# Unveiling the Role of DMAP for the Se-Catalyzed Oxidative Carbonylation of Alcohols: A Mechanism Study

**DOI:** 10.1021/acsomega.3c09813

**Published:** 2024-03-11

**Authors:** Hye Jin Lee, Seohyeon Jang, Tae Yong Kim, Jeong Woo Han, Inho Nam, Jayeon Baek, Yong Jin Kim

**Affiliations:** † Green and Sustainable Materials R&D Department, Korea Institute of Industrial Technology, Chungcheongnam-do 31056, Republic of Korea; ‡ School of Chemical Engineering and Materials Science, Department of Intelligent Energy and Industry, Department of Advanced Materials Engineering, Chung-Ang University, Seoul 06974, Republic of Korea; § Department of Materials Science and Engineering, Research Institute of Advanced Materials, Seoul National University, Seoul 08826, Republic of Korea

## Abstract

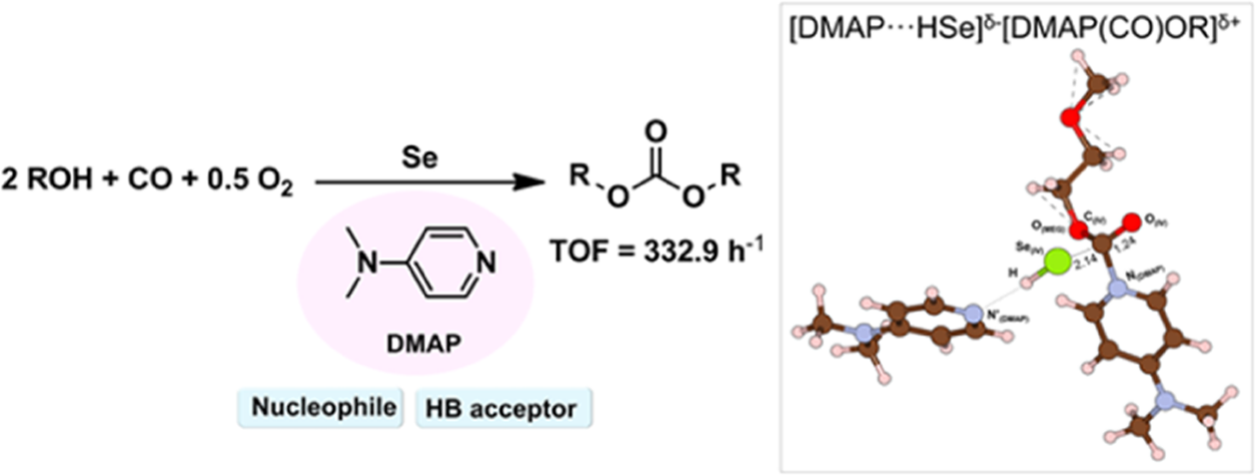

Considering the remarkable catalytic activity (160 times higher) of Se/DMAP for the oxidative carbonylation of alcohols, unveiling the role of DMAP in catalysis is highly required. We investigated DFT calculations, and the proposed intermediates were verified with in situ ATR-FTIR analysis. DFT showed that the formation of [DMAP···HSe]^δ−^[DMAP(CO)OR]^δ+^ (IV) via nucleophilic substitution of DMAP at the carbonyl group of DMAP···HSe(CO)OR is the most energetically favorable. DMAP acts as both a nucleophile and a hydrogen bond acceptor, which is responsible for its remarkable activity.

## Introduction

Dialkyl carbonates (DACs) possess significant importance in the chemical industry, including polymer, pharmaceutical, and green solvent sectors.^1−11^ In particular, DACs have found increasing applications as solvents of electrolytes in secondary Li-ion batteries.^12−18^ Therefore, considerable effort is underway to explore methods for producing DACs.^19−21^ Conventionally, the phosgene process has been preferred for industrial-scale DAC production due to its cost-effectiveness and simplicity despite the inherent hazards associated with phosgene gas.^22,23^ In the 1980s, EniChem S.p.A, as a leading company, successfully commercialized a nonphosgene process for dimethyl carbonate (DMC) production using copper halide-based catalysts in the oxidative carbonylation of methanol.^24,25^ Nevertheless, these catalysts carry some limitations, including reactor corrosion and low yield. Cobalt catalysts were studied as halide-free catalysts, but they demonstrated low catalytic activity [15.7% yield of diethyl carbonate (DEC)] and underwent deactivation.^26^

**Scheme 1:**
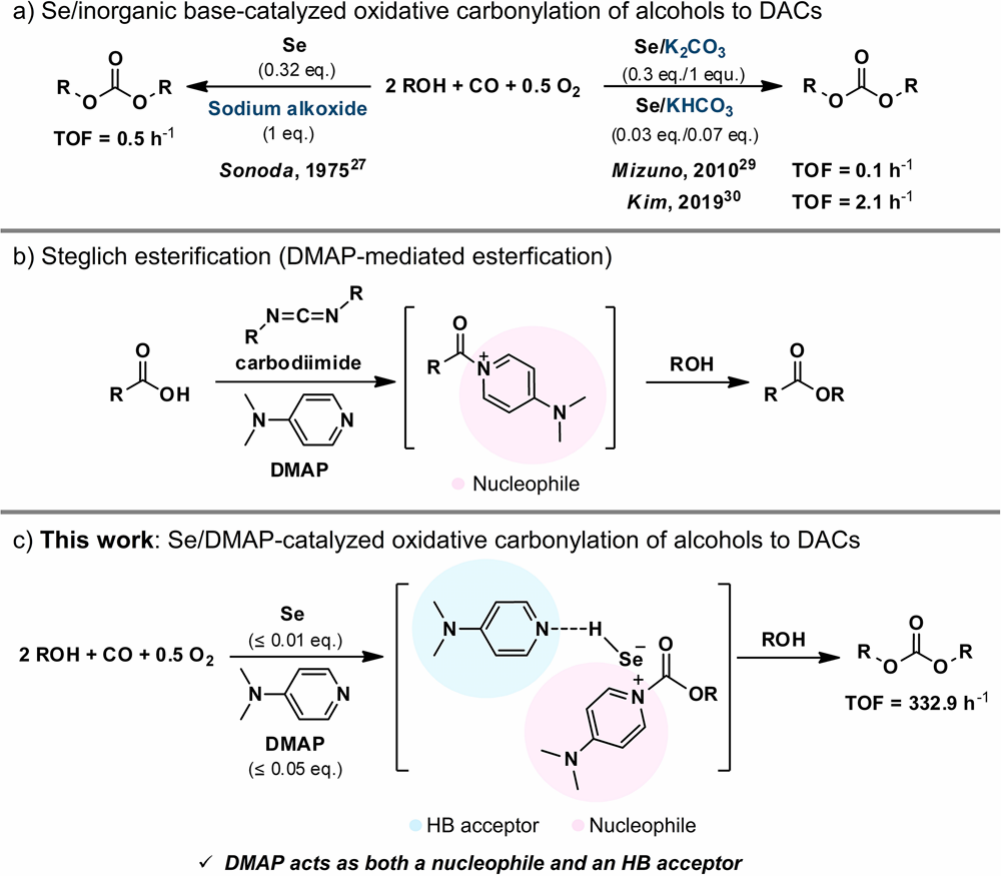
Previous and Current Work: (a) Highlights of previous studies about Se-catalyzed oxidative carbonylation. (b) Inspiration of DMAP’s role as a nucleophilic catalyst from the Steglich esterification. (c) Our proposed Se/DMAP-catalyzed oxidative carbonylation.

**Scheme 2:**
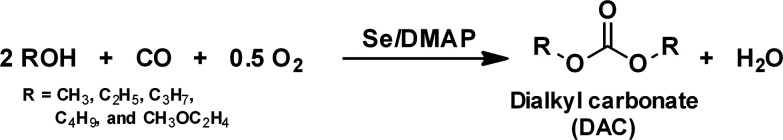
Se/DMAP-Catalyzed Oxidative Carbonylation of Alcohols

For the alternative catalyst, the Se/base catalytic system has been studied (Scheme 1a).^27−29^ In this system, the base acts as a hydrogen bond (HB) acceptor, enhancing the nucleophilicity of alcohol and thereby promoting the nucleophilic addition of alcohol to generate DACs.^29^ In our recent study, we demonstrated a catalytic system comprising Se and DMAP [DMAP = 4-(dimethylamino)pyridine], which showed a remarkable catalytic activity (60.9% yield) and recyclability (5th runs) for the oxidative carbonylation of 2-methoxyethanol (MEG) to produce bis(2-methoxyethyl carbonate) (BMEC) under mild conditions [50 °C, *n* (substrate/catalyst) = 100 or 50)].^30^ Moreover, the Se/DMAP catalytic system delivered excellent yields of DACs (over 40%) from C1–C4 alcohols even at milder reaction conditions [90 °C, *n* (substrate/catalyst) = 200], as compared to previously reported reaction conditions (120 °C) (Scheme 2).^30^

Meanwhile, there is a report on DMAP-mediated Steglich esterification (Scheme 1b), where DMAP acts as a nucleophilic catalyst.^31−35^ This mechanism clearly explains the nucleophilic substitution of DMAP at the carbonyl group of the substrate, resulting in the formation of the corresponding DMAP-based cation.^36−41^ The DMAP-based cation rapidly reacts with alcohol due to the good leaving ability of DMAP, leading to the formation of an ester compound.^36−41^

From this fact, we envisioned DMAP to show dual functionality that acts as a nucleophile as well as an HB acceptor (i.e., an intrinsic base), and this work aims to provide a precise reaction mechanism in the Se/DMAP-catalyzed oxidative carbonylation of MEG (Scheme 1c). For this purpose, various density functional theory (DFT) calculations were used to calculate energy differences as well as energy barriers for each step, and the proposed intermediates were verified with in situ ATR-FTIR analysis under the actual carbonylation conditions [50 °C, 6.12 MPa (CO/O_2_ = 7/3)]^30^ to confirm the above hypothesis.

## Results and Discussion

**Figure 1:**
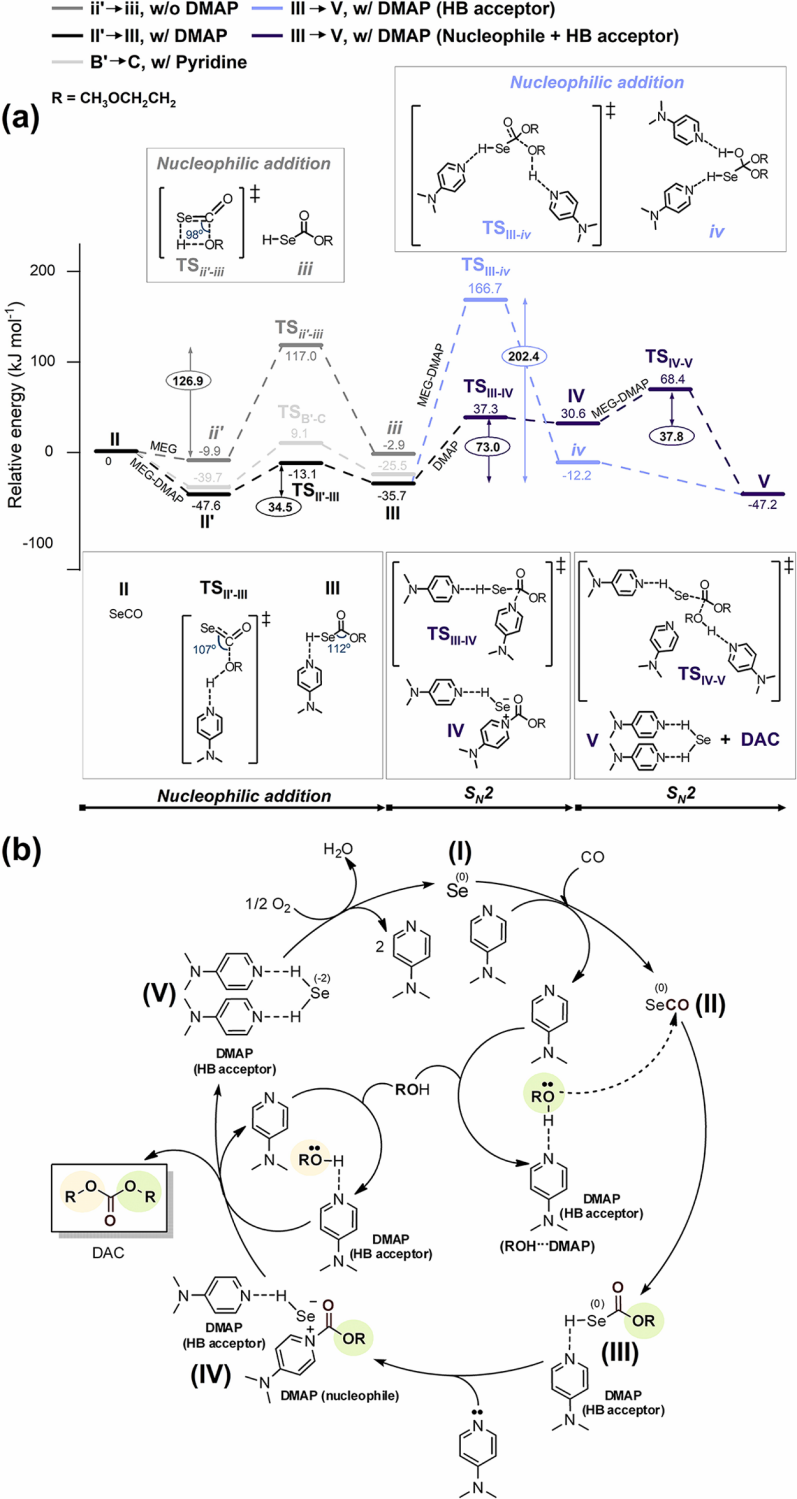
(a) Relative energy profile depending on the role of DMAP in the Se/DMAP-catalyzed oxidative carbonylation of MEG. (b) Proposed reaction mechanism of the oxidative carbonylation of alcohols to produce DACs.

Figure 1a shows the energy diagram for the Se/DMAP-catalyzed oxidative carbonylation of MEG to produce BMEC. SeCO(**II**) was chosen as the starting species (Figure 1a) in the DFT calculation due to the facile formation of **II** via the aid of a base (DMAP) with the copresence of Se(**I**) and CO.^28−30^ The first step is the approach of MEG to **II**. The effect of DMAP (or pyridine) was also considered because hydrogen-bonded MEG···DMAP has been verified by ^1^H NMR analysis in the previous work where DMAP played the role of an HB acceptor.^30^ DFT calculations also confirmed the formation of strong HB between MEG and DMAP (−41.1 kJ mol^–1^; Figure S1d). Pyridine also forms HB with MEG but with weaker interaction (−31.1 kJ mol^–1^; Figure S1e) because of the lower p*K*_a_ (5.2) compared to the p*K*_a_ of DMAP (8.9). As a result, the oxygen in the hydroxyl group of MEG···DMAP exhibited more negative charge values (−1.832 e^–^) compared to free MEG (−1.750 e^–^) and MEG···pyridine (−1.808 e^–^) (Table S1), indicating that the nucleophilicity of the oxygen of MEG is increased by the interaction with DMAP. Furthermore, the presence of DMAP led to a stronger interaction with **II**, as evidenced by their interaction energies [−9.9 (**ii**′), −39.7 (**B**′, Figure S2), and −47.6 kJ mol^–1^ (**II**′) for free MEG, MEG···pyridine, and MEG···DMAP, respectively]. Subsequently, DMAP···HSe(CO)OR (**III**) is formed through the nucleophilic addition of MEG···DMAP to the carbonyl group of **II**. Again, the presence of DMAP significantly lowers the energy barrier (**II**′ to **TS**_**II′-III**_, 34.5 kJ mol^–1^) compared to the case without DMAP (**ii**′ to **TS**_**ii′-iii**_, 126.9 kJ mol^–1^). The lower activation barrier for **TS**_**II′-III**_ is attributed to the proton shuttling [RO**H**···DMAP to DMAP···**H**Se(CO)OR] role of DMAP that can minimize distortion in the transition state. The angle of ∠Se_(SeCO)_–C_(SeCO)_–O_(MEG)_ for **TS**_**II′-III**_ is 107°, which is close to that for **III** (∠Se_(**III**)_–C_(**III**)_–O_(MEG)_ = 112°) (subset in Figure 1a and optimized structural information is presented in Figures S3b and S4). The **TS**_**ii′-iii**_, however, shows a much distorted structure [∠Se_(SeCO)_–C_(SeCO)_–O_(MEG)_ = 98° (subset in Figure 1a and optimized structural information is presented in Figure S3a)], leading to the higher activation barrier. In the case of pyridine, the energy barrier (**B**′ to TS_**B′–C**_, 48.8 kJ mol^–1^) is higher than that for DMAP. These results show that an HB acceptor is required for this reaction step, whose kinetics are affected by the p*K*_a_ value of an HB acceptor. These results also agree with our previous study that showed pyridine is less active than DMAP.^30^ In the subsequent reaction steps, the cases with pyridine and without an HB acceptor were neglected because they were found to be less active in the first reaction step than DMAP.

**Figure 2:**
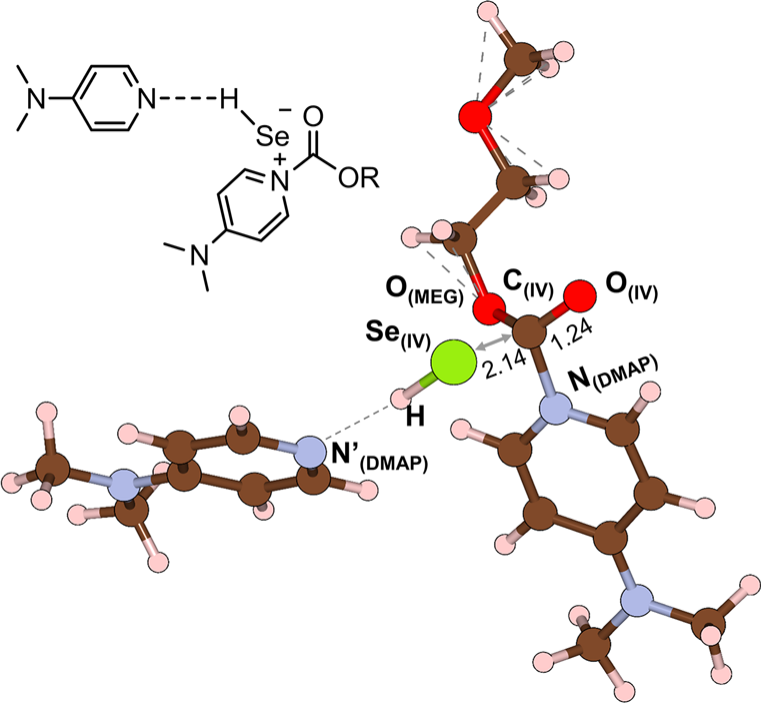
Optimized structure of intermediate **IV** with an atom label. The values without units are bond lengths (Å). The oxygen, carbon, nitrogen, and hydrogen atoms are represented in red, brown, blue, and apricot, respectively.

Intermediate **III** further reacts with the second MEG to form DAC, and there might be two plausible pathways depending on the roles of DMAP. One is a nucleophile (via **TS**_**III–IV**_) and the other is an HB acceptor (via **TS**_**III-iv**_). The former process resembles Steglich esterification,^31−35^ and the latter process is analogous to the process from **II** to **III**. Because the energy barrier for **TS**_**III-iv**_ is much higher (202.4 kJ mol^–1^) than that for **TS**_**III–IV**_ (73.0 kJ mol^–1^), here we only discuss **TS**_**III–IV**_, and a detailed discussion for **TS**_**III-iv**_ is provided in Figure S5. When DMAP acts as a nucleophile, the nitrogen atom of DMAP attacks the carbonyl carbon of **III** with the elimination of the DMAP···HSe moiety as a leaving group. In **TS**_**III–IV**_ (Figure S6a), the C_(**III**)_–N_(DMAP)_ bond is formed (1.90 Å), and the C_(**III**)_–Se_(**III**)_ bond is elongated simultaneously [2.07 Å from 1.94 Å of **III** (Figure S4)]. The charge density isosurface for **TS**_**III–IV**_ shows that a covalent C_(**III**)_–Se_(**III**)_ bond is maintained with the formation of the C_(**III**)_–N_(DMAP)_ bond, resulting in the tetrahedral coordination of carbonyl carbon (Figure S6b). This transition-state structure shows general characteristics of the S_N_2-type nucleophilic substitution reaction.^42^ The formed intermediate [DMAP···HSe]^δ−^[DMAP-(CO)OR]^δ+^ (**IV**) (Figure 2) is stabilized by the ionic interaction as confirmed by the Bader charge analysis (Table S3) and charge density isosurface (Figure S6b).

Finally, another MEG···DMAP attack on the carbonyl carbon of **IV** through **TS**_**IV–V**_ with a low energy barrier of 37.8 kJ mol^–1^ results in the generation of (DMAP···H)_2_Se (**V**) and DAC. This nucleophilic addition process is also similar to that from **II** to **III**. The reaction cycle is finished by the reaction of **V** with 1/2 O_2_, leading to the regeneration of **I** and DMAP.

Based on the DFT calculations, a plausible reaction mechanism in the Se/DMAP-catalyzed oxidative carbonylation of alcohols to DACs is illustrated in Figure 1b. **I** reacts with CO to form **II** with the aid of DMAP. MEG···DMAP reacts with **II** via nucleophilic addition, forming **III**. Nucleophilic substitution of the DMAP···HSe moiety in **III** with DMAP then takes place, and **IV** is formed. The final product, DAC, is produced by the nucleophilic addition of MEG···DMAP into **IV**, and the remaining **V** is regenerated to **I** by oxidation with O_2_.

To correlate our DFT calculations with experimental results, we conducted a series of in situ ATR-FTIR experiments from which we compared the stretching frequencies of possible intermediates with the ones from the calculations. The in situ ATR-FTIR experiments were performed under identical conditions to our catalytic reaction [50 °C, 6.12 MPa (CO/O_2_ = 7/3)^a^] in the presence of Se and DMAP.^30^ The authentic MEG, DMAP, and BMEC spectra were recorded as control samples at room temperature (Figures S8 and 3a). Notably, the ν(C=C) of the aromatics in DMAP shifted to higher values in the presence of MEG even at room temperature (from 1595, 1537, and 1514, red line, to 1606, 1541, and 1527 cm^–1^, pink line, respectively; Figure S8). Simultaneously, the frequency of the hydroxyl group in MEG shifted to lower values (from 3424 to 3405 cm^–1^), indicating elongation of the OH bond.^43,44^ This interaction between DMAP and MEG was also observed by the ^1^H NMR results, which showed a downfield shift in the hydroxyl groups (2.9−4.7 ppm; Figures S10 and S11). These findings suggest that the HB between MEG and DMAP occurs spontaneously upon mixing, which is in good agreement with previous reports.^30,45^ Therefore, we recognized that DMAP functions as an HB acceptor in the presence of alcohol, thus enhancing the nucleophilicity of the lone pair electrons on the oxygen atom in the OR group in our catalytic system.

**Figure 3:**
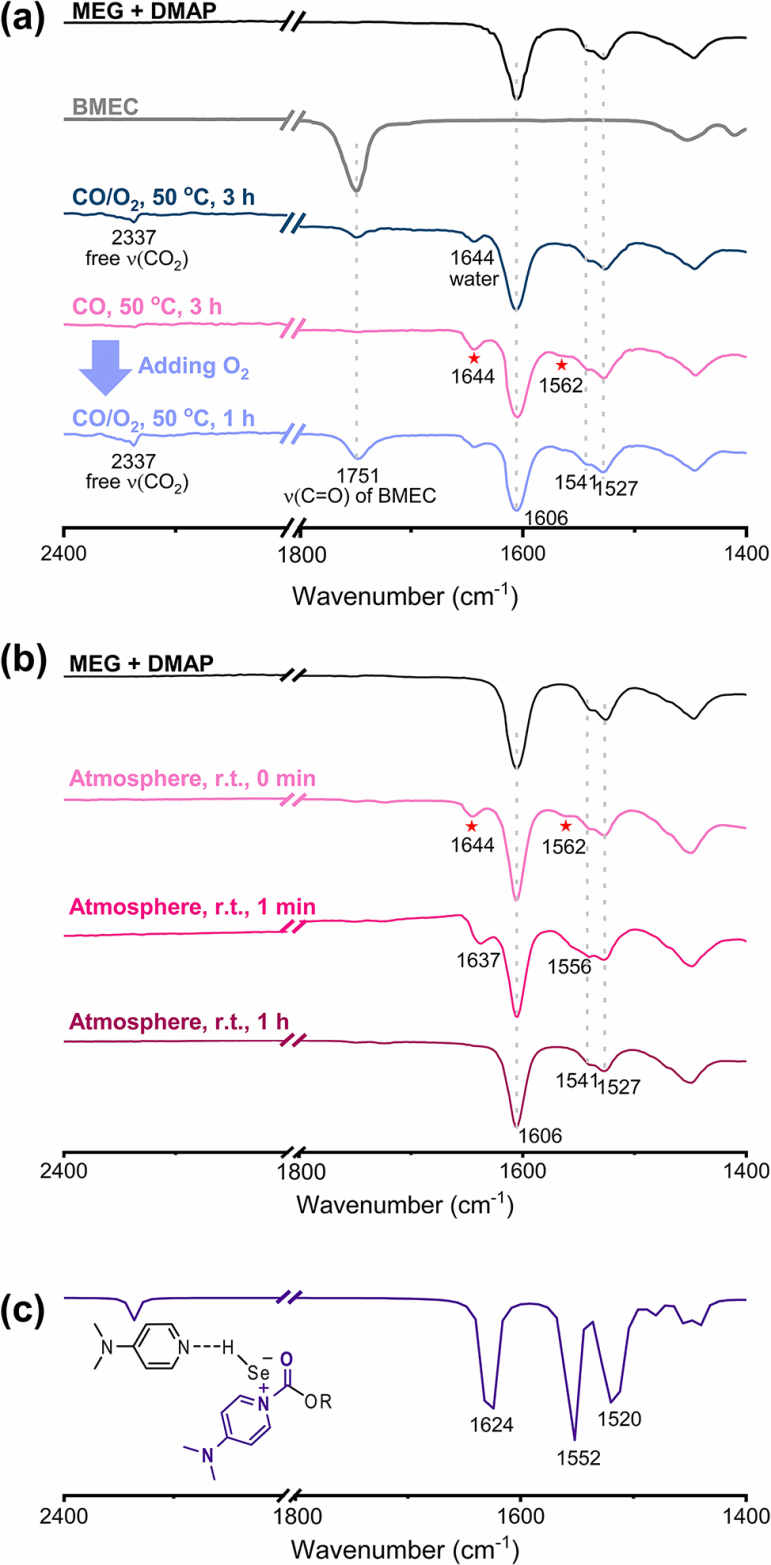
(a) In situ ATR-FTIR spectra of MEG and DMAP (black line); BMEC (gray line); MEG, Se, DMAP under CO/O_2_ (CO/O_2_ = 7/3, 6.12 MPa)^a^ at 50 °C after 3 h (dark cyan line); MEG, Se, DMAP under CO (4.28 MPa) at 50 °C after 3 h (light pink line); and MEG, Se, DMAP, and CO (4.28 MPa) at 50 °C after 3 h, followed by the addition of O_2_ (CO/O_2_ = 7/3, 6.12 MPa)^a^ at 50 °C after 1 h (pale blue line). (b) ATR-FTIR spectrum of MEG and DMAP (black line); and ex situ time-specific ATR-FTIR spectra of solids obtained from the overnight reaction of MEG and Se in DMAP under CO (6.12 MPa) at 50 °C from 0 min to 1 h (pinkish lines). (c) Calculated IR spectrum of intermediate IV obtained using Gaussian16 software at the 6-311++G(2d,p) functional level.

Upon the introduction of CO/O_2_ (7/3, 6.12 MPa)^a^ into the solution in the ATR-FTIR reaction cell and heating to 50 °C, new peaks at 2337, 1751, and 1644 cm^–1^ were observed (Figure 3a, dark cyan line). The peak at 2337 cm^–1^ corresponds to the free CO_2_ originating from CO and O_2_, and the peak at 1751 cm^–1^ is clear evidence of the carbonyl group formation in BMEC. Meanwhile, at this moment, we believed that the peak at 1644 cm^–1^ was attributed to the H_2_O generated during the reaction (see Scheme 2 and the water peak in Figure S12).

Since we did not obtain any peaks responsible for intermediates, we assumed that it might be due to the rapid catalytic cycle in the presence of O_2_. Thus, we conducted a series of in situ ATR-FTIR experiments under only CO gas (4.28 MPa) to partially quench the catalytic cycle partially (locking). Surprisingly, a peak at 1644 cm^–1^ was again observed, which was supposed not to appear without O_2_. Simultaneously, a new broad peak appeared at 1562 cm^–1^ (Figure 3a, light pink line). Upon introduction of the O_2_ (1.84 MPa)^a^ into the same ATR-FTIR reaction cell (unlocking), the carbonyl peak of BMEC reappeared along with free CO_2_ (pale blue line in Figure 3a and blue lines in Figure S14).

To gain further insight into the observed peaks at 1644 and 1562 cm^–1^, a wet solid (MEG did not evaporate completely) obtained from the overnight reaction of Se and DMAP in MEG under a CO (6.12 MPa) at 50 °C was analyzed using ex situ time-specific ATR-FTIR. As shown in the light pink line of Figure 3b, the peaks at 1644 and 1562 cm^–1^ were again obtained together with the original peaks of MEG···DMAP (1606, 1541, and 1527 cm^–1^). Upon exposure to air within 1 min, these peaks shifted to 1637 and 1556 cm^–1^ and completely disappeared after 1 h (Figure 3b, pinkish lines). These observations suggest that the peak at 1644 cm^–1^ did not solely originate from H_2_O but rather might be responsible for an important Se-based intermediate in the Se/DMAP-catalyzed oxidative carbonylation of MEG. To end this, the peaks at 1644 and 1562 cm^–1^ were compared with the calculated peaks through DFT calculations. Interestingly, the calculated IR spectrum of intermediate **IV** (purple line, 1624 and 1552 cm^–1^; Figure 3c) closely matches with the values experimentally measured at 1644 and 1562 cm^–1^ (light pink lines of Figure 3a,b).

**Figure 4:**
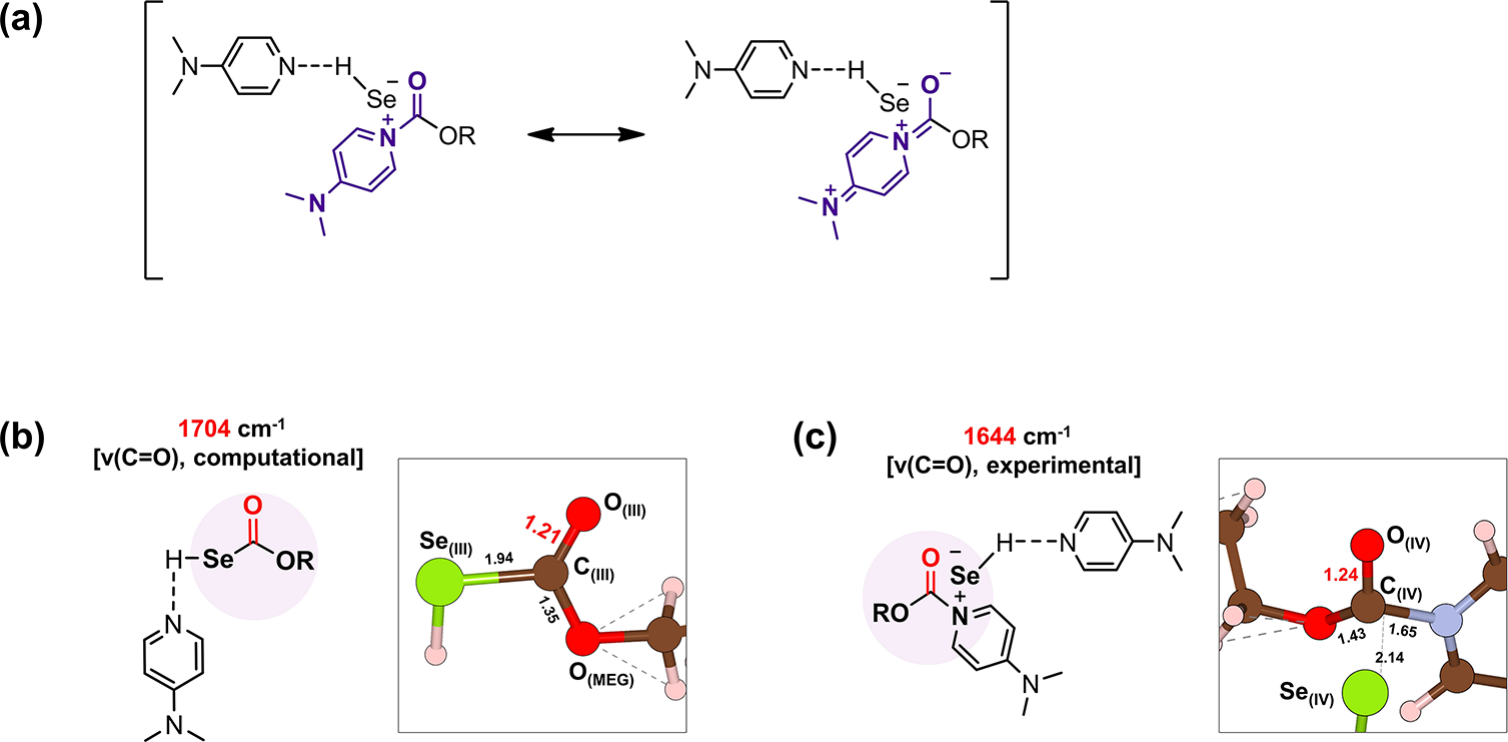
(a) Resonance structures of intermediate **IV**. Structural information on (b) **III** and (c) **IV**. The values without units are bond lengths (Å). The oxygen, carbon, nitrogen, and hydrogen atoms are represented in red, brown, blue and apricot, respectively.

Briefly speaking, the peaks at 1644 and 1562 cm^–1^ can be attributed to the resonance structure of **IV** where 1644 cm^–1^ corresponds to C=O and 1562 cm^–1^ corresponds to C=C as illustrated in Figure 4a (see also the Supporting Video clip for the stretching mode of intermediate **IV** obtained from DFT calculations).^46^ Additionally, the peak at 1644 cm^–1^ corresponding to the carbonyl of **IV** is supported by an elongated C_(**IV**)_=O_(**IV**)_ bond length (1.24 Å, Figure 4c), in contrast to the C_(**III**)_=O_(**III**)_ bond length of 1.21 Å (Figure 4b). This elongation is indicative of a red shift from the peak at 1704 cm^–1^ corresponding to ν(C=O) of **III** (Figures 4b and S15). The rapid disappearance of the peaks at 1644 and 1562 cm^–1^ upon exposure to air might be due to the fact that intermediate **IV** is very unstable. Overall results strongly support that DMAP acts not only as an HB acceptor but also as a nucleophile in the Se/DMAP-catalyzed oxidative carbonylation of MEG, which is the main reason for the high activity.

Meanwhile, material characterizations of the used Se were conducted to confirm whether the used Se has a difference from fresh Se.^47^ In XPS, upon curve fitting the Se 3d spectra, both fresh and used Se peaks could be assigned to elemental Se^0^ (Figure S16).^48−50^ We also conducted FTIR analysis to verify that the used Se does not contain any organic selenium compounds after the catalytic cycle, which is responsible for possible changes in the selenium valence. In the FTIR analysis, no peaks corresponding to organic compounds were found (Figure S17). These observations indicate the regeneration of Se^0^ during the catalytic cycle.

In summary, our study provided a comprehensive investigation into the mechanistic details of the Se/DMAP-catalyzed oxidative carbonylation of alcohols to produce DACs. Based on the DFT calculations and spectroscopic data, the proposed pathway generating intermediate **IV** was found to be a critical step and energetically favorable. Based on these findings, we suggested a plausible reaction mechanism. Notably, DMAP exhibited dual-functional behavior, acting as both a nucleophile and HB acceptor, which is responsible for the remarkable productivity of DACs in the Se-catalyzed oxidative carbonylation of alcohols.

## Experimental Section

### Materials

Selenium (99.5%, ∼325 mesh), DMAP (+98%), MEG (98%), and CDCl_3_ (99.8%, contains 0.03% v/v TMS) were purchased from Alfa Aesar. CO and O_2_ (99.95% pure) were obtained from Gong-Dan Industrial Gas Co., South Korea. All chemicals were used without further purification.

### Characterizations

The NMR spectra of DMAP, MEG, and HB in MEG···DMAP were obtained using a Bruker Spectrospin 300 MHz spectrometer at 25 °C with tetramethylsilane (TMS) as an internal standard. Before the measurements, the samples were dissolved in CDCl_3_. X-ray photoelectron spectroscopy (XPS) was studied to characterize the oxidation states of the fresh and used Se using a Thermo Fisher instrument equipped with an Al K-α radiation source (energy range from 100 to 3k eV). To compensate for the charging effects, the Se 3d spectra have been corrected to the C 1s spectra set to 284.6 eV.

### Procedure for In Situ ATR-FTIR under Mixture Gas

The in situ ATR-FTIR analysis under mixed gas conditions was conducted in a golden gate reaction cell (Specac) with a diamond window. MEG (21 mmol), Se (1.5 mmol), and DMAP (7.5 mmol) were added to the reaction cell and then flushed three times and pressurized with mixed gas (CO/O_2_ = 7/3) at 5.78 MPa (to make 6.12 MPa at 50 °C). The reaction cell was placed in a Nicolet 6700 FTIR spectrometer (Thermo Fisher Scientific) and heated to 50 °C, and the FTIR spectra were recorded at a specific time.

### Procedure for In Situ ATR-FTIR under CO Followed by Adding O_2_

The experimental details were the same as those above, except for using CO as the flushing and pressurizing gas until a certain pressure was attained (Thermo Fisher Scientific). After 3 h, O_2_ gas was added to the reactor at 50 °C until pressure reached 6.12 MPa. The measurements were monitored in real time, and the FTIR spectra were recorded at specific times.

### Procedure for Ex Situ ATR-FTIR

The Se carbonyl complex was investigated by ex situ ATR-FTIR using a Nicolet iS10 FTIR spectrometer (Thermo Fisher Scientific) equipped with a SMART MIRacle accessory (ZnSe window). Before the measurement, the carbonylation of MEG (65 mmol) using Se (5 mmol) and DMAP (10 mmol) was conducted in a pressurized reactor under a CO atmosphere (6.12 MPa) at 50 °C overnight. After completion of the CO treatment, the wet solid (with MEG) was collected and promptly subjected to ATR-FTIR analysis.

### Reaction Energy Barrier and Charge Density Analysis

DFT calculations were performed using the Vienna ab initio simulation package (VASP).^51−54^ Electronic structures were optimized using the Perdew–Burke–Ernzerhof (PBE) exchange–correlation functional and generalized gradient approximation (GGA) with projector-augmented wave pseudopotential (PAW).^55−58^ A cutoff energy of 400 eV and a 1 × 1 × 1 Monkhorst–Pack mesh for *k*-point sampling were used. Electronic optimization was performed until the energy variation during the self-consistent field (SCF) cycle was less than 1 × 10^–5^ eV, and all geometries were optimized until the residual force was less than 0.05 eV/Å. The charge density isosurface level is set as 1.11 e^–^ bohr^–3^.

The climbing image nudged elastic band (CI-NEB) method in conjunction with the dimer method was employed to locate the transition-state structures and calculate the activation energy barrier. The NEB method was first used to determine the transition-state structure over the minimum energy path between the reactant and product. Five or seven images were generated from the linear interpolation between the reactant and product images and were used as an initial estimate to find the minimum energy path. The major molecules governing each reaction step were reflected in NEB calculations. This method can reduce computational errors by eliminating minor molecules; thus, the behavior of the major molecules can be precisely investigated. The same cutoff energy, *k*-point sampling, and SCF energy criteria were used, but a force criterion of 0.1 eV/Å was employed. The obtained transition-state structures were further refined using the dimer method with a force criterion of 0.06 eV/Å. The charge density of the optimized structures was explored using Bader charge analysis.^59−61^

### DFT-Based Vibrational Frequency Calculations

Vibrational frequency calculations were performed by using the Gaussian16 program. All calculations were conducted within DFT formalism using Becke’s three-parameter with the gradient-corrected Lee, Yang, and Parr correlation (B3LYP) hybrid functional and a 6-311G basis set supplemented by two diffuse functions.^62−64^ Two sets of d polarization functions were added to the heavy atoms, and one set of p polarization functions was added to the hydrogen atoms.

## Supporting Information Available

Experimental details, computational calculation data, FTIR data, and NMR data (PDF)
The animation of the stretching mode of intermediate **IV** (MP4)

## Notes

^a^Caution: A ratio of CO/O_2_ less than 7/3 can lead to an explosion. Handling it necessitates careful attention and proper protective measures.
