# Supplementary material for: Unveiling the Role of DMAP for the Se-Catalyzed Oxidative Carbonylation of Alcohols: A Mechanism Study
Source: ACS Omega. 2024 Mar 11;9(11):13200–7. doi: 10.1021/acsomega.3c09813 (PMC10955696; doi:10.1021/acsomega.3c09813)
Supplement: Supplementary file 1 — ao3c09813_si_001.pdf [file ao3c09813_si_001.pdf]

Unveiling the Role of DMAP for the Se-Catalyzed Oxidative Carbonylation of Alcohols: A Mechanism Study

Hye Jin Lee,‡[a] Seohyeon Jang,‡[b] Tae Yong Kim,‡[c] Jeong Woo Han,[c] Inho Nam,*[b] Jayeon Baek*[a] and Yong Jin Kim,*[a]

[a]*Green and Sustainable Materials R&D Department, Korea Institute of Industrial Technology, 89 Yangdaegiro-gil, Ipjang-myeon, Seobuk-gu, Cheonan-si, Chungcheongnam-do, 31056, Republic of Korea. E-mail: yjkim@kitech.re.kr (Y.J. Kim) and jbaek@kitech.re.kr (J. Baek)*

[b]*School of Chemical Engineering and Materials Science, Department of Intelligent Energy and Industry, Department of Advanced Materials Engineering, Chung-Ang University, 84 Heukseok-ro, Dongjak-gu, Seoul, 06974, Republic of Korea. E-mail: inhonam@cau.ac.kr*

[c]*Department of Materials Science and Engineering, Research Institute of Advanced Materials, Seoul National University, 1, Gwanak-ro, Gwanak-gu, Seoul, 08826, Republic of Korea*

‡These authors contributed equally.

* To whom correspondence may be addressed: Yong Jin Kim (yjkim@kitech.re.kr), Jayeon Baek (jbaek@kitech.re.kr), Inho Nam (inhonam@cau.ac.kr)

Table of content

DFT calculations 1

Structure of MEG, DMAP, pyridine, MEG⋯DMAP and MEG⋯pyridine ……………………..….....… 1

Bader charge analysis ....….....… 2

Energy barrier investigation of oxidative carbonylation by pyridine ....… 3

Structure comparison between $TS_{\textit{ii'-iii}}$, $TS_{\mathbf{II'\text{-}III}}$, and $TS_{\mathbf{B'\text{-}C}}$ by DFT calculations 4

Structure investigation on intermediate **III** by DFT calculations 5

Mechanism study on $TS_{\mathbf{III}\text{-}\textit{iv}}$ when DMAP acts only as an HB acceptor 6

Structure comparison between $TS_{\mathbf{III\text{-}IV}}$ and $TS_{\mathbf{III}\text{-}\textit{iv}}$ by DFT calculations 7

Investigation on intermediate **IV** via charge density and structure analysis 8

Experimental Results...11

Hydrogen bond formation of (MEG and DMAP) using FT-IR and ^{1}H NMR analysis 11

Mechanism study using in situ and ex situ ATR-FTIR spectra 14

Calculated IR spectra of intermediates using DFT calculations 17

XPS spectra of fresh and used Se 18

FT-IR spectra of fresh and used Se 19

Reference 20

DFT calculations

Structure of MEG, DMAP, pyridine, MEG⋯DMAP and MEG⋯pyridine

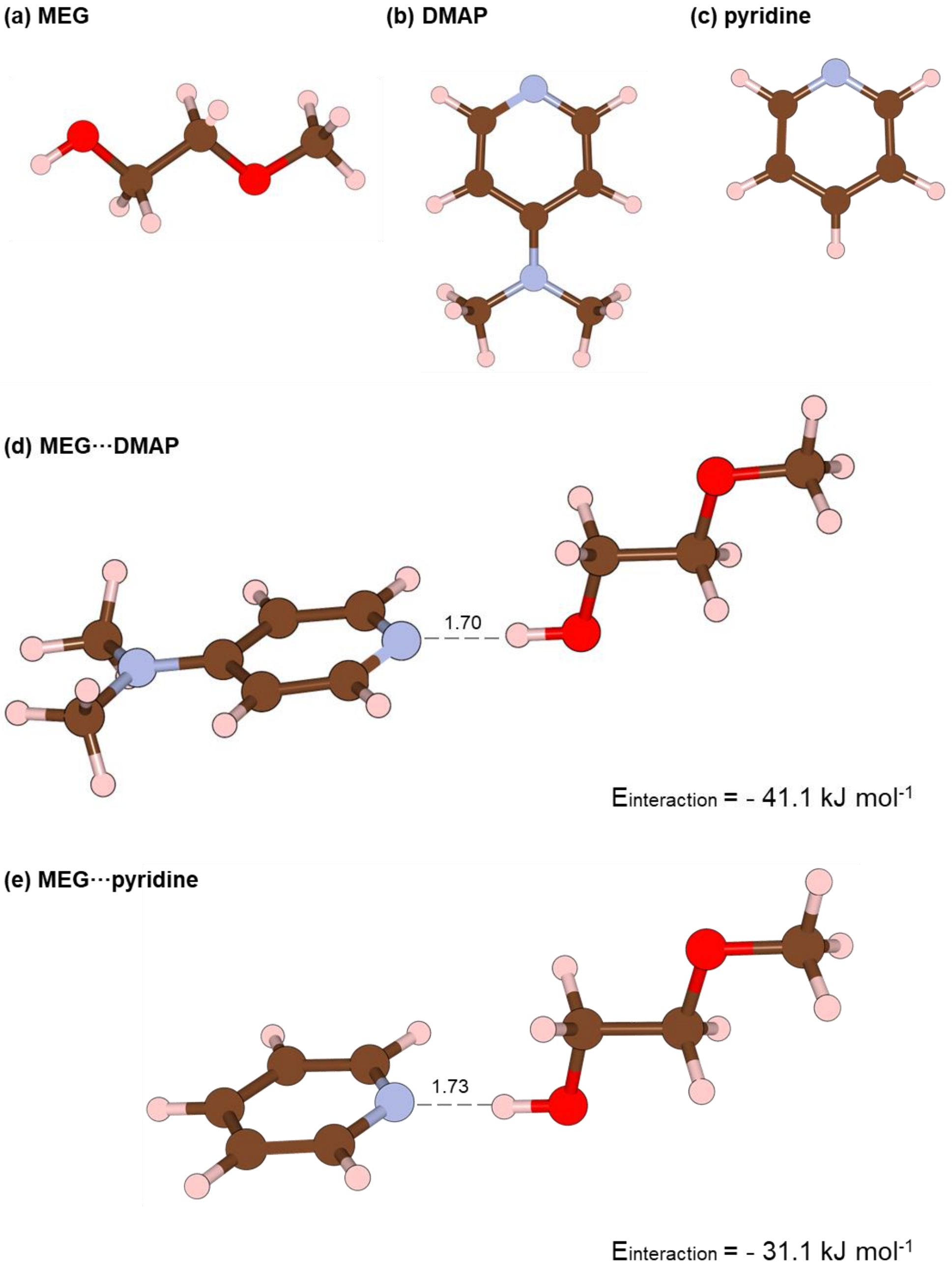

Figure S1. Structural information of (a) MEG, (b) DMAP, (c) pyridine, (d) MEG⋯DMAP and (e) MEG⋯pyridine. The values without units are bond lengths (Å). The oxygen, carbon, nitrogen, and hydrogen atoms are represented in red, brown, blue and apricot, respectively. The interaction energy between MEG and HB acceptor (DMAP or pyridine) is calculated by the following equation. Einteraction = E(MEG⋯ HB acceptor) – E(MEG) – E(HB acceptor)

Bader charge analysis

Table S1. Bader charge analysis on the oxygen in hydroxyl group of MEG

Complex	Number of electrons in O atom (e^-)	Charge[a]
Free MEG	7.750	-1.750
MEG⋯DMAP	7.832	-1.832
MEG⋯pyridine	7.808	-1.808

[a] Charge = 6 – number of electrons in O atom

Energy barrier investigation of oxidative carbonylation by pyridine

To study the difference between the effects of DMAP and pyridine whose pK_a values are different, calculations for the oxidative carbonylation of MEG by pyridine was carried out. The energy barrier for **$TS_{B'-C}$** is 48.8 kJ mol^{-1} (Figure S2a), which is higher than the case of DMAP (34.5 kJ mol^{-1}, Figure 1a). The results demonstrate that pyridine is less active in oxidative carbonylation reactions than DMAP, and this is in line with our previous research (The yield of BMEC and TOF value with the use of DMAP are 60.9 % and 32.5 h^{-1}, while 0.6 % and 0.3 h^{-1} with the use of pyridine).[1]

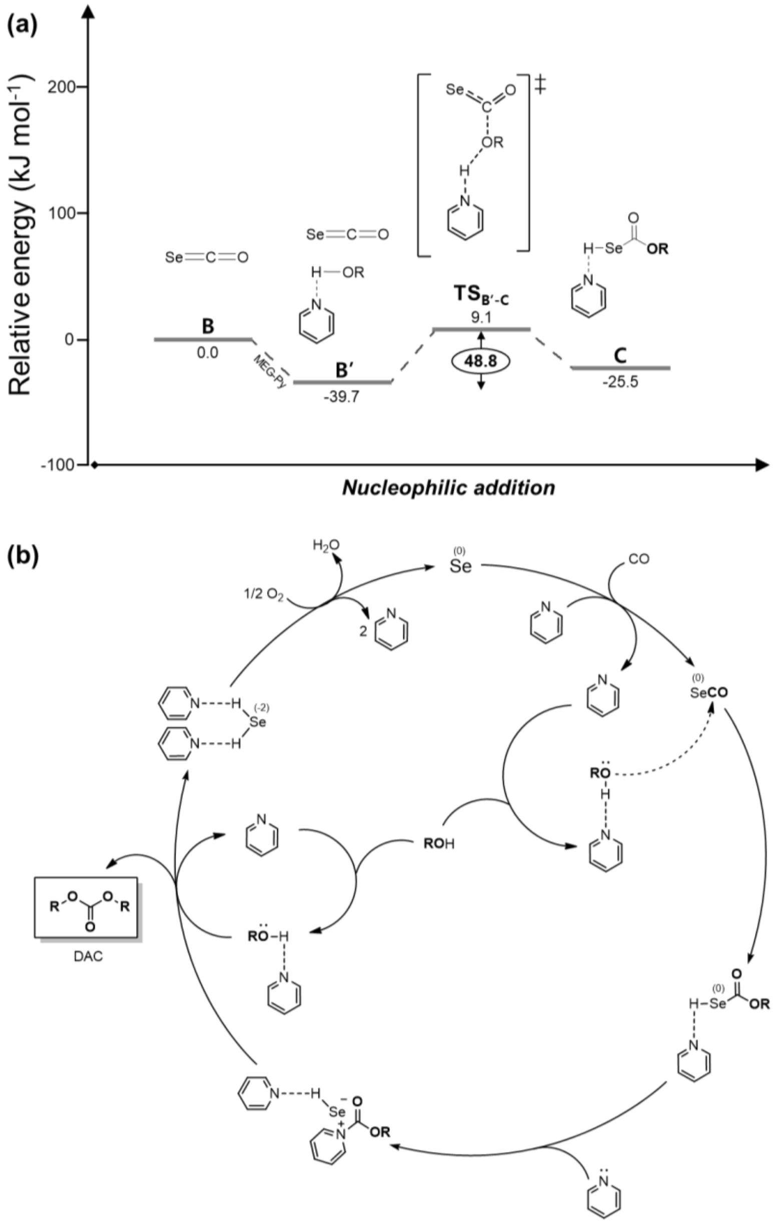

Figure S2. (a) Relative energy profile when pyridine follows the same reaction mechanism proposed in Fig. 1b. (b) Reaction mechanism in which pyridine acts as a catalyst.

Structure comparison between $TS_{ii'\text{-}iii}$, $TS_{II'\text{-}III}$, and $TS_{B'\text{-}C}$ by DFT calculations

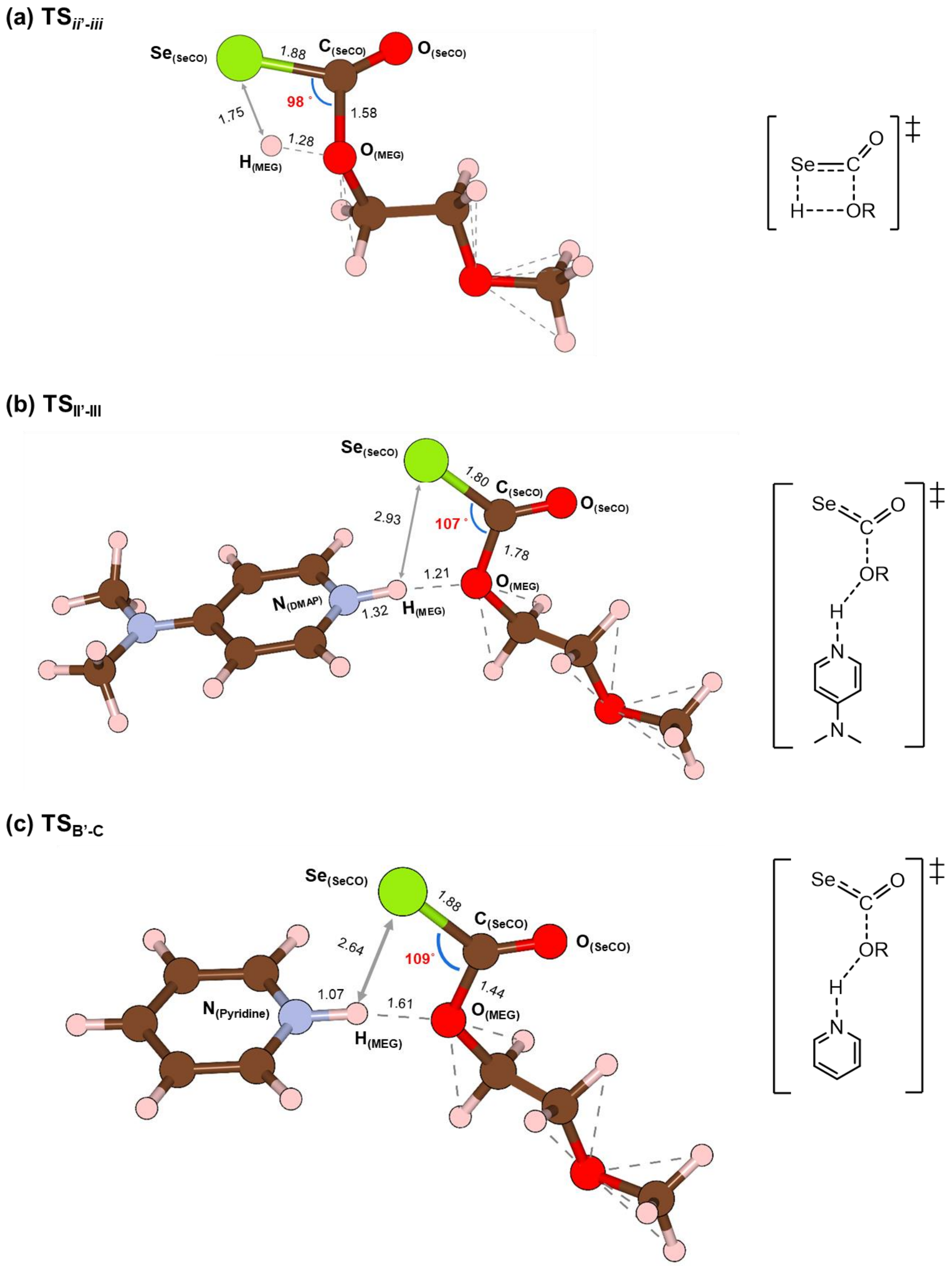

Figure S3. Structural information of (a) $\mathbf{TS}_{ii'\text{-}iii}$, (b) $\mathbf{TS}_{II'\text{-}III}$ and (c) $\mathbf{TS}_{B'\text{-}C}$. The values without units are bond lengths (Å). The oxygen, carbon, nitrogen, selenium and hydrogen atoms are represented in red, brown, blue, green and apricot, respectively.

Structure investigation on intermediate III by DFT calculations

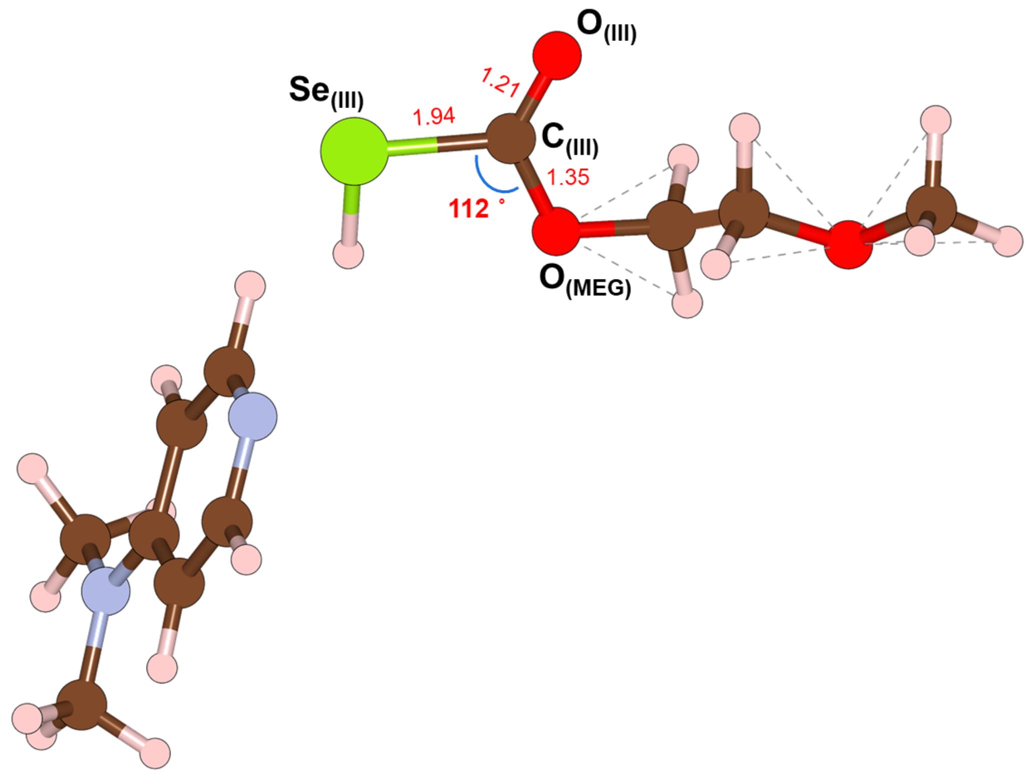

Figure S4. Optimized structure of intermediate **III**. The values without units are bond lengths (Å). The oxygen, carbon, nitrogen, selenium and hydrogen atoms are represented in red, brown, blue, green and apricot, respectively.

Mechanism study on $TS_{III\text{-}iv}$ when DMAP acts only as an HB acceptor

When DMAP acts as an HB acceptor, MEG···DMAP approaches to the carbonyl carbon of **III** as illustrated in Figure S5. The nucleophilic addition of MEG···DMAP on the carbonyl carbon of **III** through $TS_{III\text{-}iv}$ proceeded with high energy barrier of 202.4 kJ mol^{-1}, forming intermediate ***iv*** (Figure 1a). The distances of $C_{(III)}$–$O_{(MEG)}$ and $C_{(III)}$–$O_{(III)}$ are 1.89 and 1.29 Å, indicating the approach of MEG···DMAP to the intermediate III and elongation of carbonyl group, which accompanies dissociation of $O_{(MEG)}$–$H_{(MEG)}$ bond (1.36 Å) (Figure S6d). The elongation of the carbonyl group indicates breaking of p bond to the O, resulting in the formation of an alkoxide. Protonation of the alkoxide occurs with angle strains i.e., $O_{(III)}$–$C_{(III)}$–$O_{(MEG)}$, $C_{(III)}$–$O_{(III)}$–$H_{(MEG)}$, and $C_{(III)}$–$O_{(MEG)}$–$H_{(MEG)}$ are 89, 85, and 60°, respectively (Figure S6d).

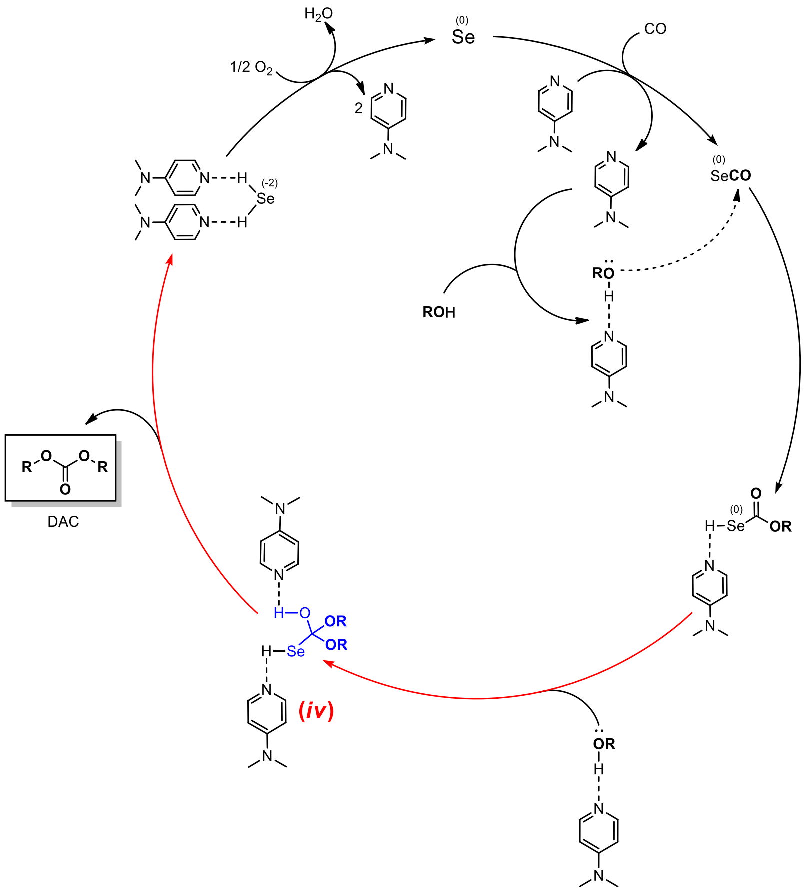

Figure S5. Catalytic cycle of the reaction when DMAP acts only as an HB acceptor. The red arrow steps are different from those represented in Fig. 1b.

Structure comparison between $TS_{III\text{-}IV}$ and $TS_{III\text{-}iv}$ by DFT calculations

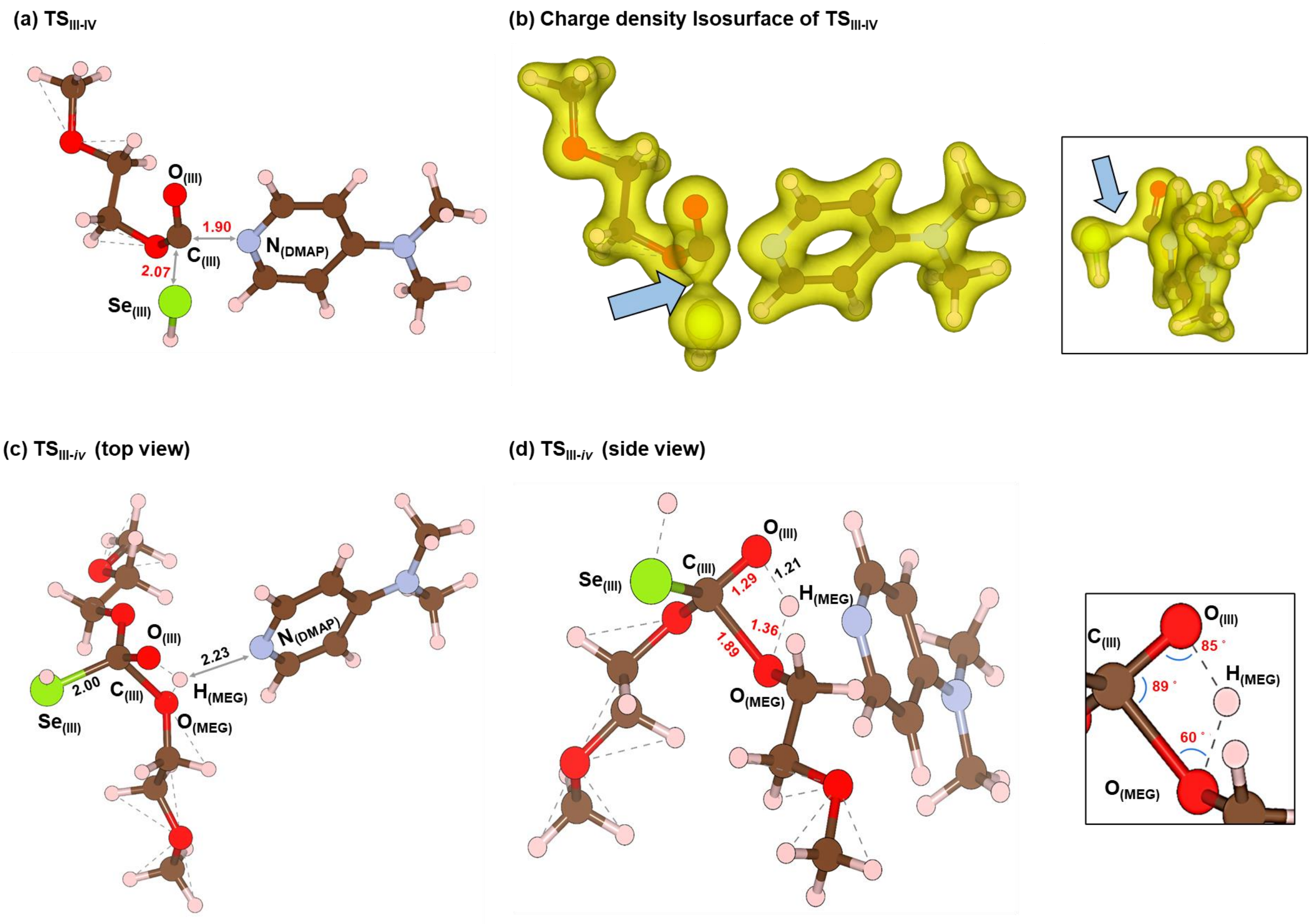

Figure S6. (a) Structural information, and (b) charge density isosurface of $\mathbf{TS_{III\text{-}IV}}$ with side view inset (isosurface level is set to be 1.11 e^{-} $bohr^{-3}$)**.** The light blue arrow indicates the maintaining $C_{(III)}$–$Se_{(III)}$ bond. Structural information of (c) $\mathbf{TS_{III\text{-}\mathit{iv}}}$ (top view), and (d) $\mathbf{TS_{III\text{-}\mathit{iv}}}$ (side view)**.** The values without units are bond lengths (Å). The inset next to the $\mathbf{TS_{III\text{-}\mathit{iv}}}$ (side view) is an expanded image of $O_{(III)}$-$C_{(III)}$-$O_{(MEG)}$-$H_{(MEG)}$ region with bond angles. To investigate the nucleophilic behavior solely, the DMAP molecule stabilizing HSe moiety was eliminated in $\mathbf{TS_{III\text{-}IV}}$ and $\mathbf{TS_{III\text{-}\mathit{iv}}}$. The oxygen, carbon, nitrogen, selenium, and hydrogen atoms are represented in red, brown, blue, green, and apricot, respectively.

Investigation on intermediate IV via charge density and structure analysis

The structure of intermediate **IV** exhibits a distorted tetrahedral structure as shown in Figure S7a. The bond length of the $C_{(IV)}$–$O_{(IV)}$ bond of the carbonyl group in **IV** is 1.24 Å, which is longer than that of the C=O double bond (1.21 Å, Figure S4) in **III**. The bond length of the $Se_{(IV)}$–$C_{(IV)}$ bond is 2.14 Å, which is longer than that observed in intermediate **III** (1.94 Å, Figure S4). According to the Bader charge analysis, the charge of the DMAP···HSe moiety is partially negative at -0.12 e^-, while the DMAP-(CO)OR moiety is partially positive at +0.12 e^-. The charge density isosurface also shows the ionic interaction (light blue arrow in Figure S7b). This demonstrates that the non-localized electrons were conjugated over intermediate **IV**. The detailed structural information and charge density assigned to each moiety are shown in Tables S2 and S3.

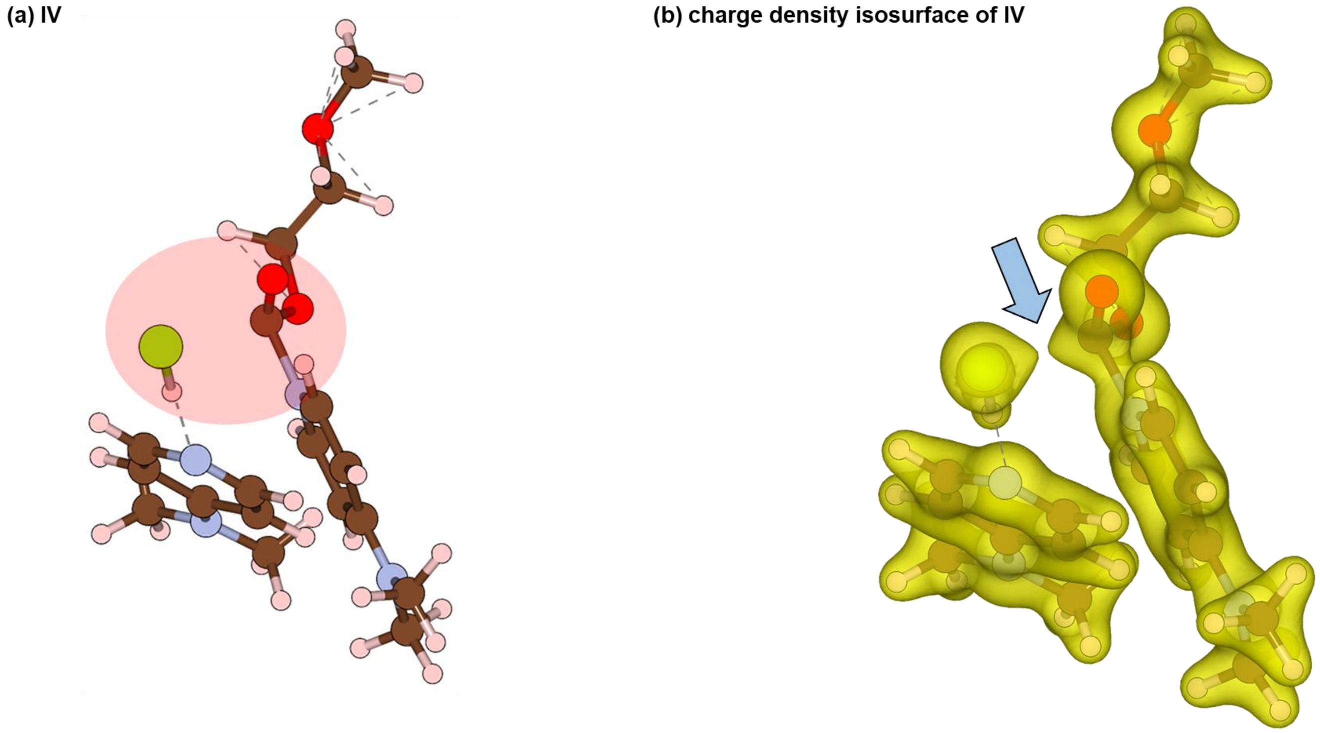

Figure S7. (a) Structural information, and (b) charge density isosurface of intermediate **IV** (isosurface level is set to be 1.11 e^- bohr^{-3}). A distorted tetrahedral structure of **IV** is highlighted with a red circle. The values without units are bond lengths (Å). The oxygen, carbon, nitrogen, selenium and hydrogen atoms are represented in red, brown, blue, green and apricot, respectively.

Table S2. Selected distances and angles for intermediate **IV**

Bond Length (Å)		Bond Angles (˚)	
$C_{(\mathbf{IV})}$–$N_{(DMAP)}$	1.65	$O_{(\mathbf{IV})}$–$C_{(\mathbf{IV})}$–$O_{(MEG)}$	122
$C_{(\mathbf{IV})}$–$O_{(\mathbf{IV})}$	1.24	$O_{(\mathbf{IV})}$–$C_{(\mathbf{IV})}$–$N_{(DMAP)}$	112
$C_{(\mathbf{IV})}$–$O_{(MEG)}$	1.43	$O_{(\mathbf{IV})}$–$C_{(\mathbf{IV})}$–$Se_{(\mathbf{IV})}$	111
$C_{(\mathbf{IV})}$–$Se_{(\mathbf{IV})}$	2.14	$O_{(MEG)}$–$C_{(\mathbf{IV})}$–$N_{(DMAP)}$	98
$Se_{(\mathbf{IV})}$–H	1.50	$O_{(MEG)}$–$C_{(\mathbf{IV})}$–$Se_{(\mathbf{IV})}$	109
H–N’$_{(DMAP)}$	2.41	$N_{(DMAP)}$–$C_{(\mathbf{IV})}$–$Se_{(\mathbf{IV})}$	103

Table S3. Bader charge analysis results for intermediate **IV**

DMAP (nucleophile)	C	N	H	Total electrons (e^-)
Calculated electrons	26.31	12.34	8.96	47.61
Valence electrons	28	10	10	48
Differences	1.69	-2.34	1.04	0.39
(CO)OR	C	O	H	
Calculated electrons	12.85	21.79	6.62	41.27
Valence electrons	16	18	7	41
Differences	3.15	-3.79	0.38	-0.27
Partial charge of DMAP-(CO)OR Moiety				**+0.12**

DMAP (HB acceptor)	C	N	H	Total electrons (e^-)
Calculated electrons	26.26	12.39	9.34	47.99
Valence electrons	28	10	10	48
Differences	1.74	-2.39	0.66	0.01
HSe	H	Se		
Calculated electrons	1.07	6.61		7.13
Valence electrons	1	6		7
Differences	-0.07	-0.06		-0.13
Partial charge of DMAP···HSe Moiety				**-0.12**

FT-IR and NMR

Hydrogen bond formation of (MEG and DMAP) using FT-IR and ^{1}H NMR analysis

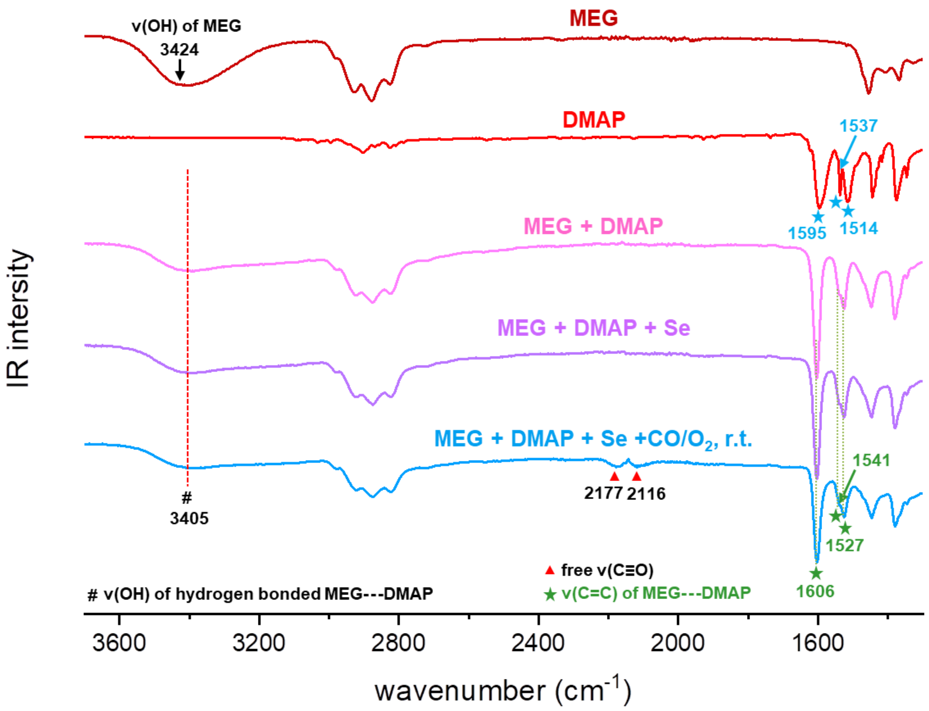

Figure S8. In situ ATR-FTIR spectra of MEG (chocolate line); DMAP (red line); MEG and DMAP (pink line); MEG, DMAP, and Se (pale purple line); MEG, DMAP, and Se, after the introduction of CO/O_2 at room temperature (light blue line).

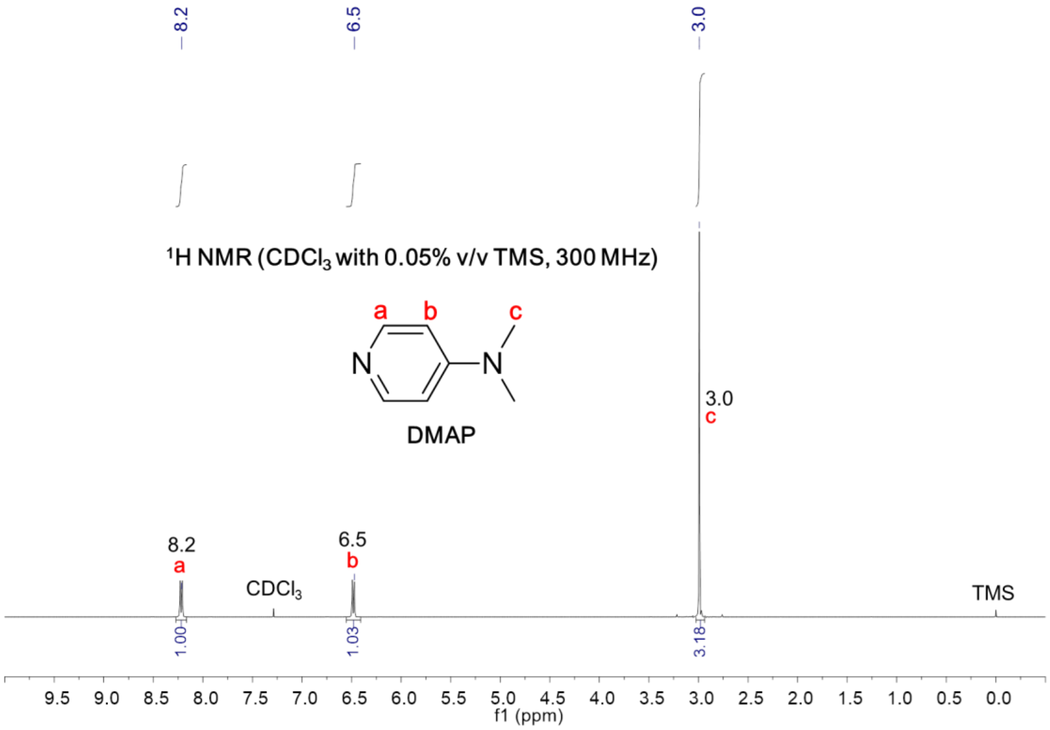

Figure S9. ^{1}H NMR spectrum (300 MHz, $CDCl_3$) of **DMAP**.

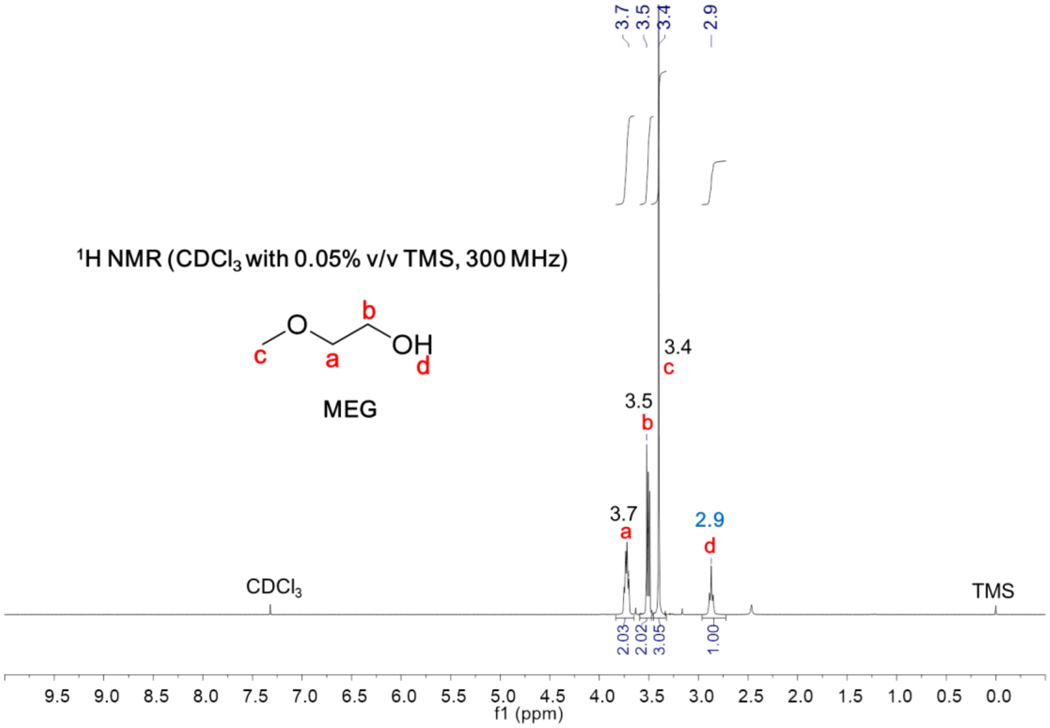

Figure S10. ^{1}H NMR spectrum (300 MHz, $CDCl_3$) of **MEG**.

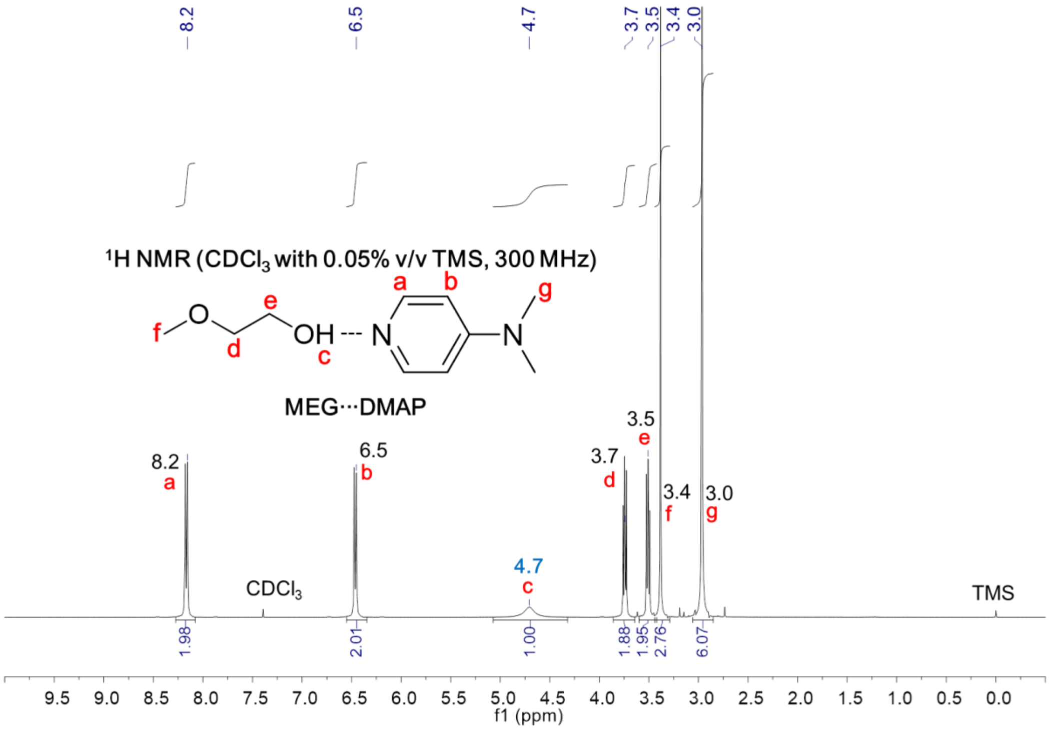

Figure S11. ^{1}H NMR spectrum (300 MHz, $CDCl_3$) of **MEG···DMAP**

Mechanism study using in situ and ex situ ATR-FTIR spectra

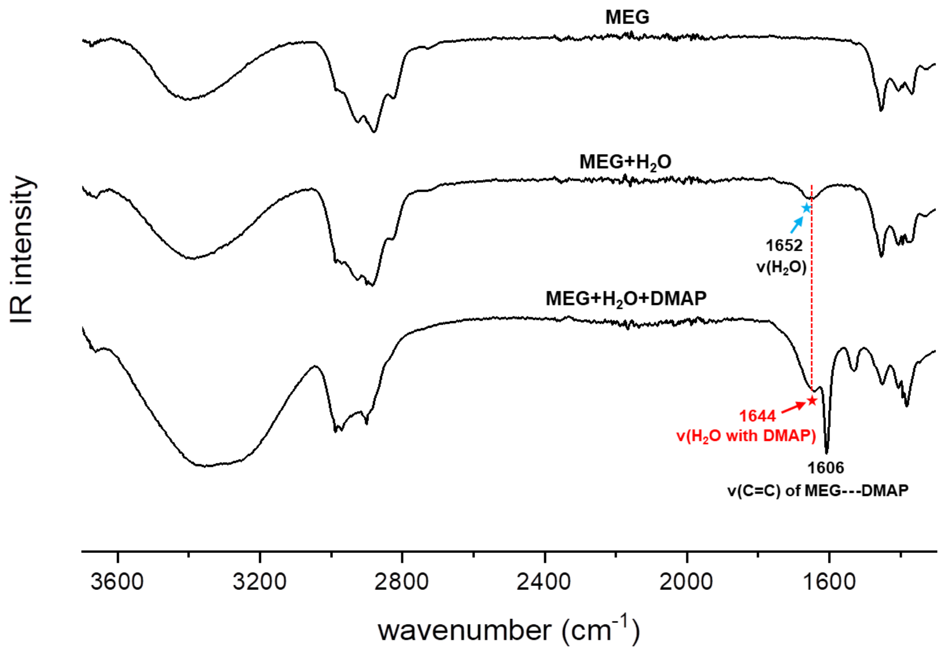

Figure S12. ATR-FTIR spectra of MEG; MEG + H_2O; and MEG + H_2O + DMAP.

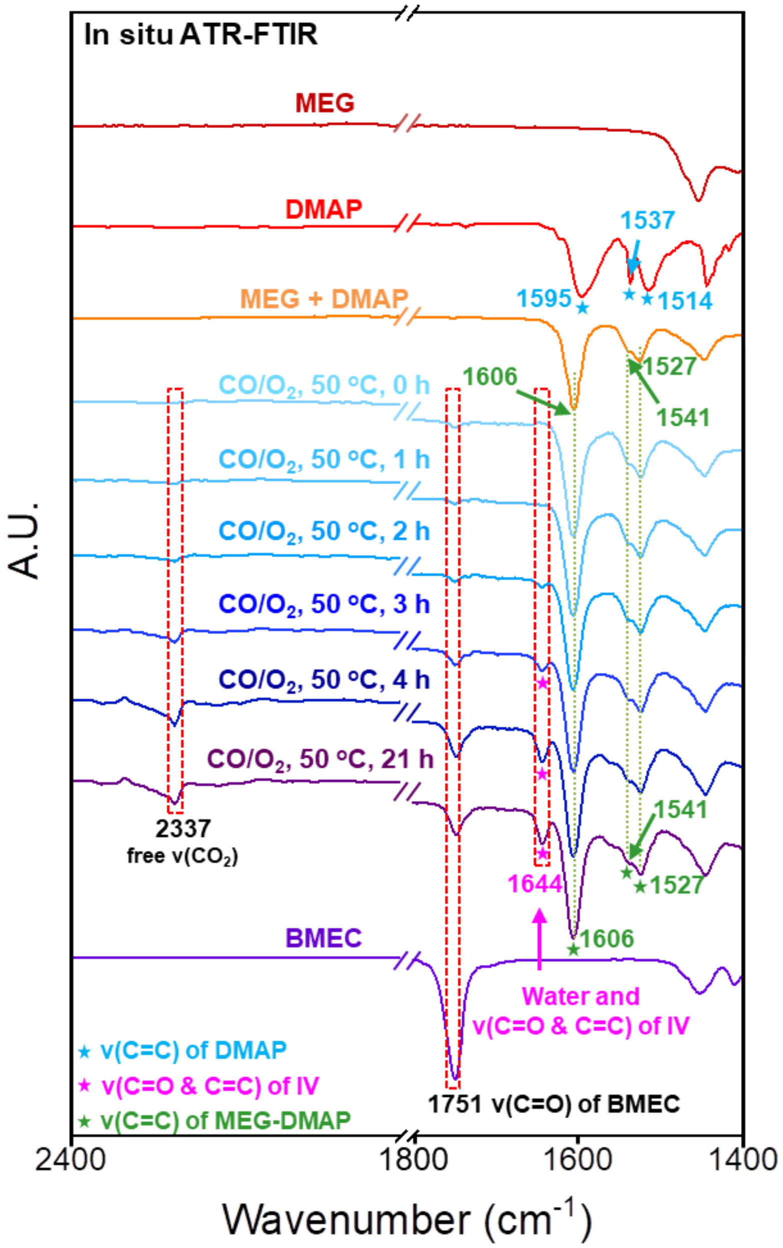

Figure S13. In situ time-specific ATR-FTIR spectra of MEG, Se, DMAP, and CO/O_2 (CO/O_2 = 7/3, 6.12 MPa) at 50 °C.

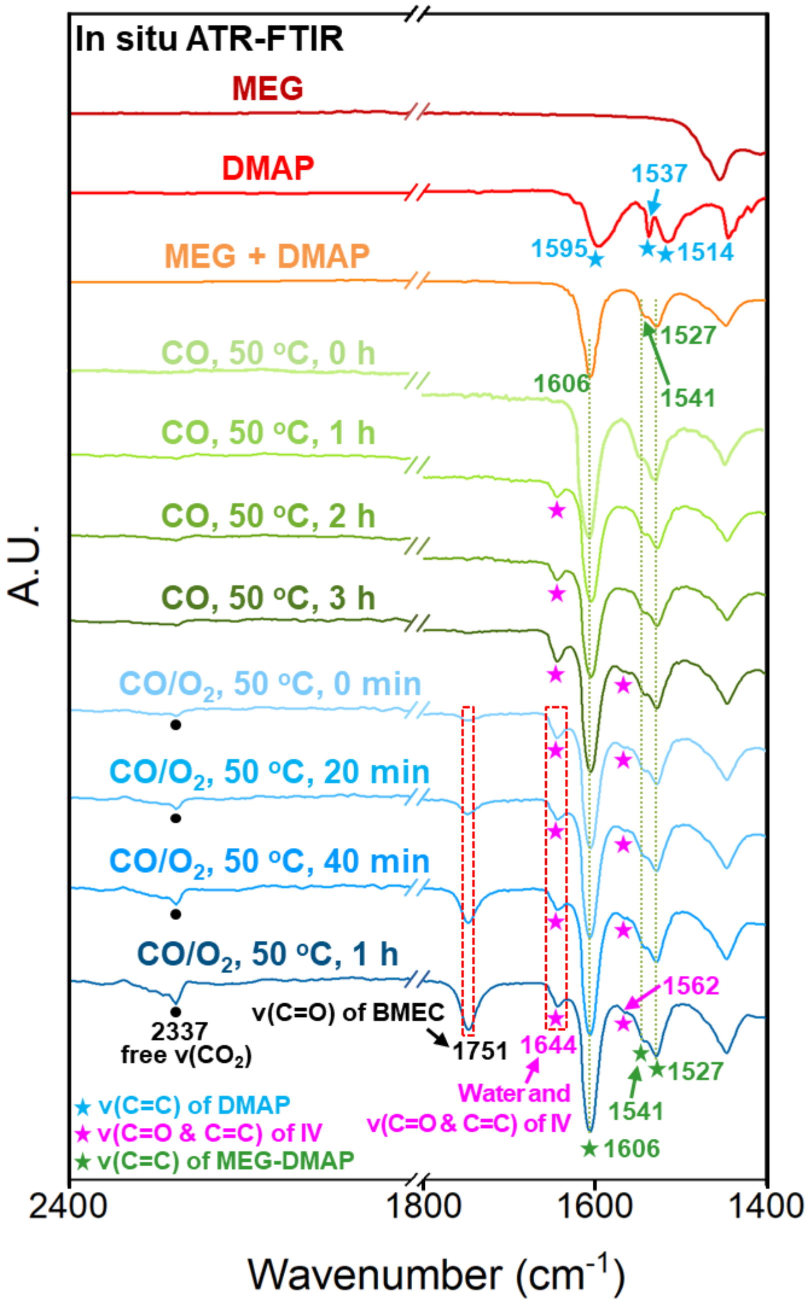

Figure S14. In situ time-specific ATR-FTIR spectra of MEG, Se, DMAP, and CO (4.28 MPa) at 50 °C (green lines), followed by the addition of O_2 (CO/O_2 = 7/3, 6.12 MPa) at 50 °C (blue lines).

Calculated IR spectra of intermediates using DFT calculations

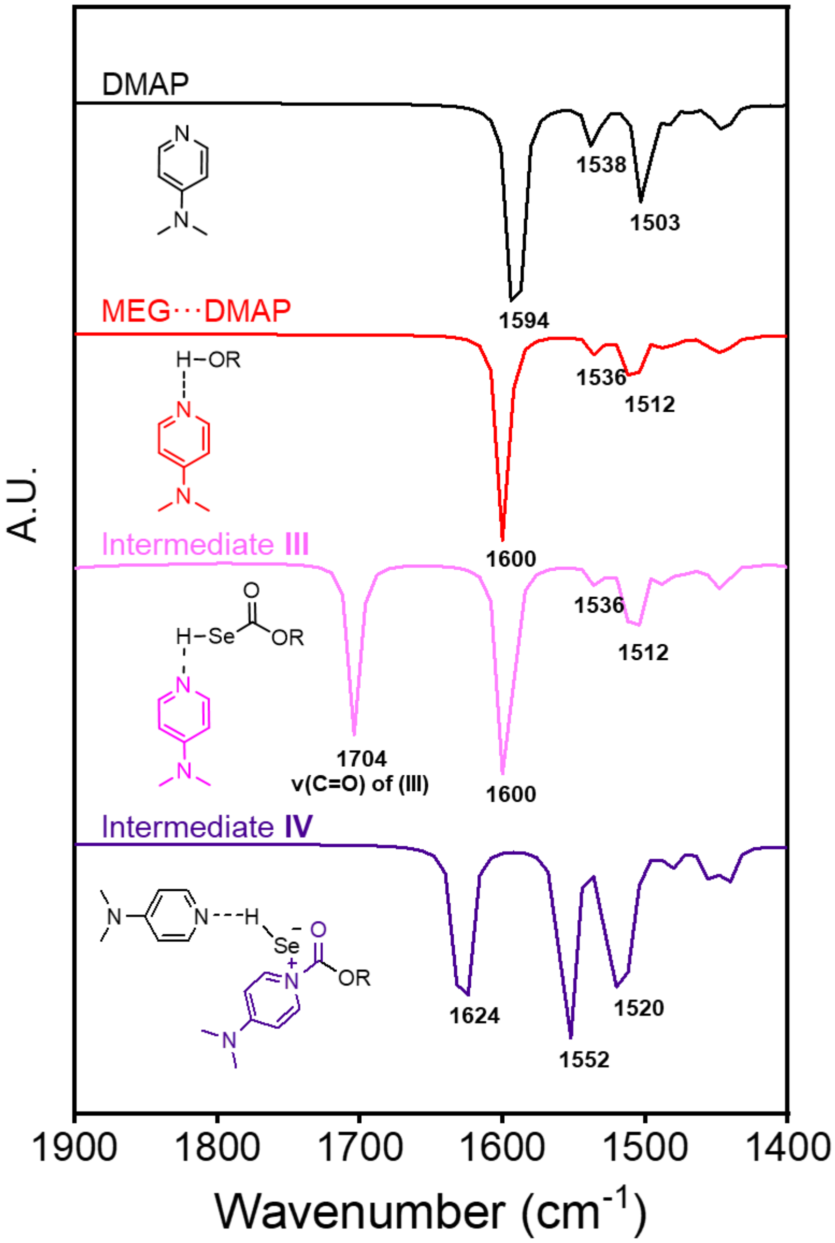

Figure S15. Calculated IR spectra of free DMAP (black), MEG + DMAP (red), and intermediates **III** (pink) and **IV** (purple) obtained using the Gaussian16 software at the 6-311++G(2d,p) functional level. The animation of stretching mode of intermediate **IV** can be observed in Supplementary video clip.

XPS spectra of fresh and used Se

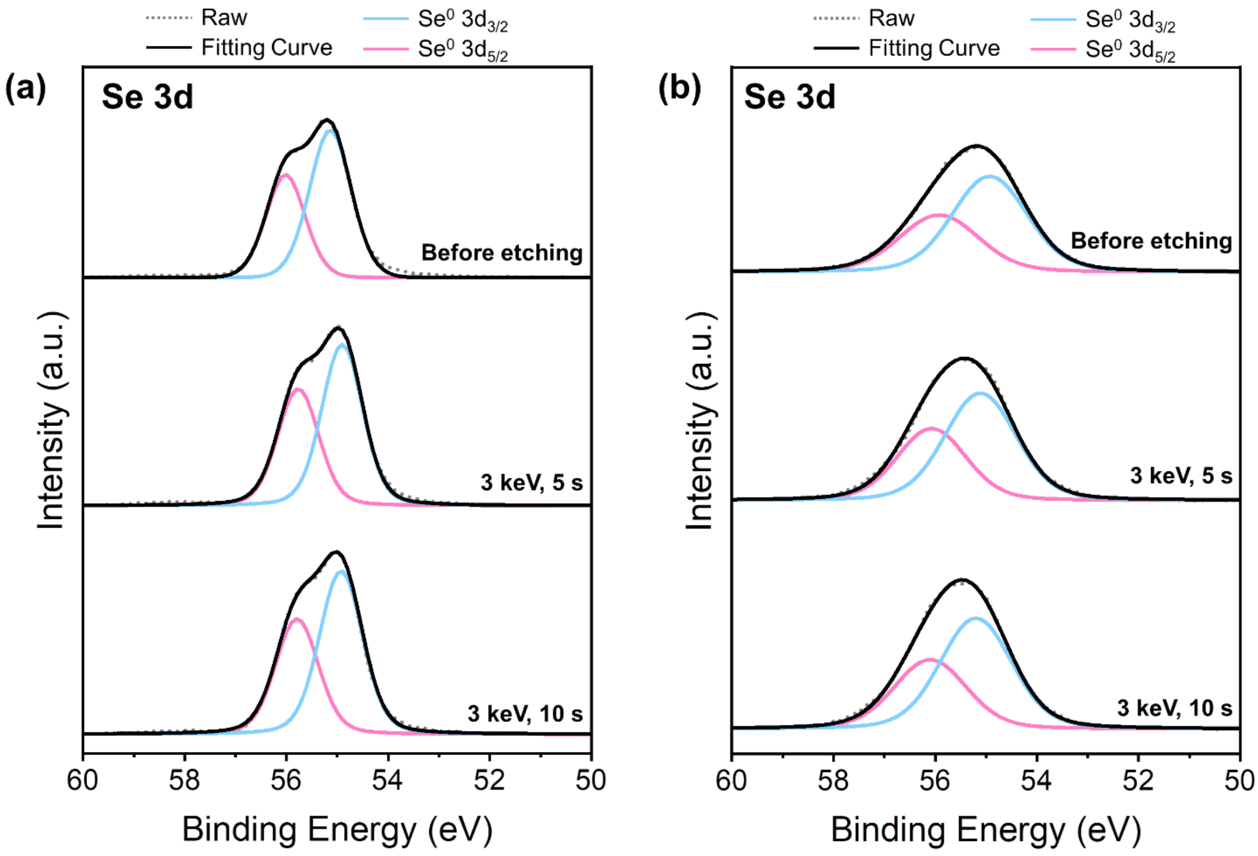

Figure S16. XPS spectra of Se 3d of (a) fresh and (b) used Se.

As shown in Figure S16, the Se 3d spectrum shape of used Se differed from that of fresh Se. However, by curve fitting the Se 3d spectra, the binding energies of Se $3d_{3/2}$ and Se $3d_{5/2}$ in the fresh Se were measured as 55.8 ± 0.1 and 55.0 ± 0.1 eV, respectively (Figure S16a). Furthermore, for used Se, the corresponding binding energies of Se $3d_{3/2}$ and Se $3d_{5/2}$ were determined as 56.1 ± 0.1 and 55.2 ± 0.1 eV, respectively (Figure S16b). According to Version 5.0 of the National Institute of Standards and Technology (NIST) X-ray Photoelectron Spectroscopy Database (SRD 20), the elemental Se exhibits Se 3d peaks at 55.4 ± 0.7.[2] Therefore, both fresh and used Se peaks are assigned to elemental Se^0.

FT-IR spectra of fresh and used Se

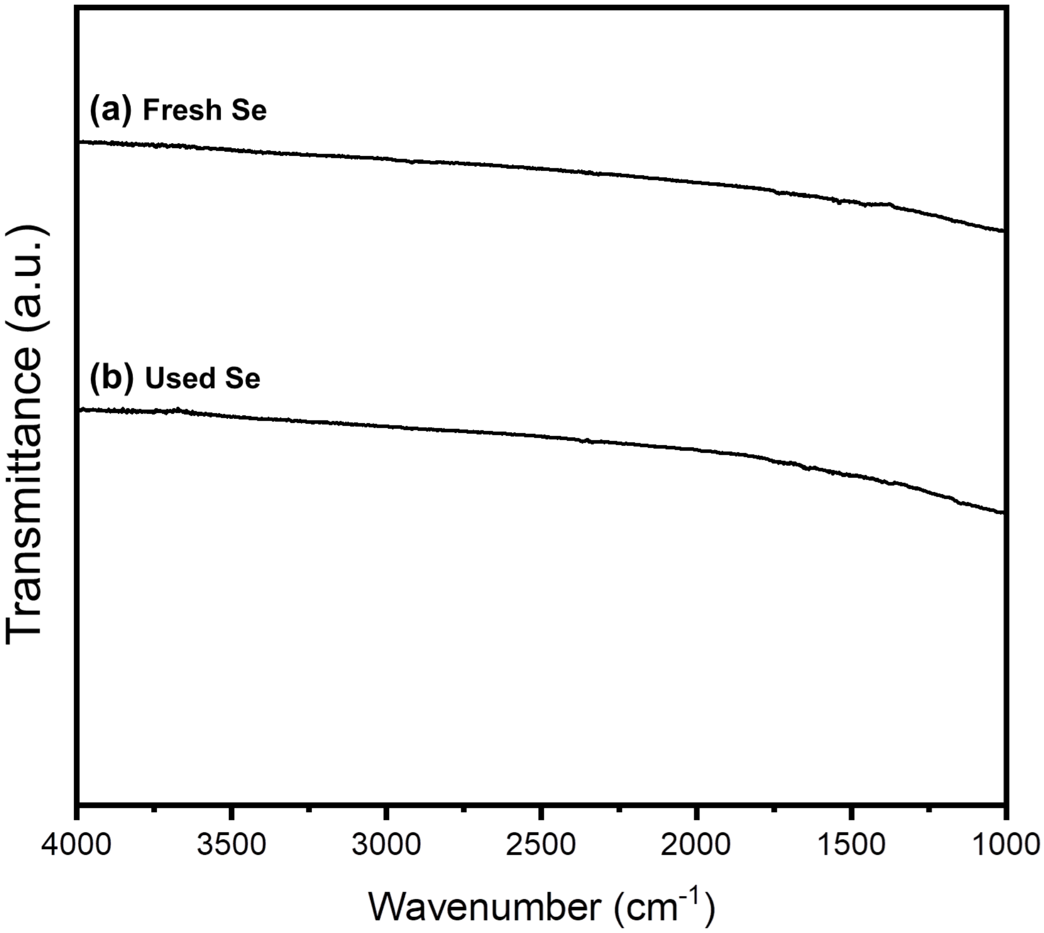

Figure S17. FT-IR spectra of (a) fresh and (b) used Se.

Reference

(1) Lee, H. J.; Nguyen, T. T.; Tran, A. V.; Kim, H. S.; Suh, Y.-W.; Baek, J.; Kim, Y. J., Engineering pK_a value of 3° amine for enhanced production of dialkyl carbonate via Se-catalyzed oxidative carbonylation. *Journal of Industrial and Engineering Chemistry* **2023,** *123*, 140-149.

(2) https://srdata.nist.gov/xps/selEnergyType.aspx, accessed February 5, 2024.
